# Supplementary material for: Molecular architecture of the human tRNA ligase complex
Source: eLife. 2021 Dec 2;10:e71656. doi: 10.7554/eLife.71656 (PMC8668186; doi:10.7554/eLife.71656)
Supplement: Transparent reporting form [file elife-71656-transrepform1.pdf]

eLIFE
1st Floor
24 Hills Road
Cambridge CB2 1JP, UK
P 01223 855340
W elifesciences.org
T @elife

eLife's transparent reporting form

We encourage authors to provide detailed information *within their submission* to facilitate the interpretation and replication of experiments. Authors can upload supporting documentation to indicate the use of appropriate reporting guidelines for health-related research (see EQUATOR Network), life science research (see the BioSharing Information Resource), or the ARRIVE guidelines for reporting work involving animal research. Where applicable, authors should refer to any relevant reporting standards documents in this form.

If you have any questions, please consult our Journal Policies and/or contact us: editorial@elifesciences.org.

Sample-size estimation

- You should state whether an appropriate sample size was computed when the study was being designed
- You should state the statistical method of sample size computation and any required assumptions
- If no explicit power analysis was used, you should describe how you decided what sample (replicate) size (number) to use

Please outline where this information can be found within the submission (e.g., sections or figure legends), or explain why this information doesn't apply to your submission:

No explicit power analysis was used.

Replicates

- You should report how often each experiment was performed
- You should include a definition of biological versus technical replication
- The data obtained should be provided and sufficient information should be provided to indicate the number of independent biological and/or technical replicates
- If you encountered any outliers, you should describe how these were handled
- Criteria for exclusion/inclusion of data should be clearly stated
- High-throughput sequence data should be uploaded before submission, with a private link for reviewers provided (these are available from both GEO and ArrayExpress)

Please outline where this information can be found within the submission (e.g., sections or figure legends), or explain why this information doesn't apply to your submission:

For the cross-linking/mass spectrometry analysis (Figure 2), two independent technical replicates were performed for each of the protein samples (5-subunit full tRNA-LC and 4-subunit core tRNA-LC). The same stock of purified protein complex was diluted into two independent samples for the cross-linking reaction and further MS analysis. The peptide spectrum matches between the two runs were merged (union).

Statistical reporting

eLife Sciences Publications, Ltd is a limited liability non-profit non-stock corporation incorporated in the State of Delaware, USA, with company number 5030732, and is registered in the UK with company number FC030576 and branch number BR015634 at the address 1st Floor, 24 Hills Road, Cambridge CB2 1JP | August 2014

- Statistical analysis methods should be described and justified
- Raw data should be presented in figures whenever informative to do so (typically when N per group is less than 10)
- For each experiment, you should identify the statistical tests used, exact values of N, definitions of center, methods of multiple test correction, and dispersion and precision measures (e.g., mean, median, SD, SEM, confidence intervals; and, for the major substantive results, a measure of effect size (e.g., Pearson's r, Cohen's d)
- Report exact p-values wherever possible alongside the summary statistics and 95% confidence intervals. These should be reported for all key questions and not only when the p-value is less than 0.05.

Please outline where this information can be found within the submission (e.g., sections or figure legends), or explain why this information doesn't apply to your submission:

For the cross-linking/mass spectrometry experiment, the cross-linked peptide spectral matches were scored by a linear discriminant score and the threshold of 30 was chosen by inspection of spectral quality. This threshold corresponds to a decoy database-based calculation of <5% false discovery rate at the identification level calculated according to Walzthoeni, 2012 Nat Methods.

(For large datasets, or papers with a very large number of statistical tests, you may upload a single table file with tests, Ns, etc., with reference to sections in the manuscript.)

Group allocation

- Indicate how samples were allocated into experimental groups (in the case of clinical studies, please specify allocation to treatment method); if randomization was used, please also state if restricted randomization was applied
- Indicate if masking was used during group allocation, data collection and/or data analysis

Please outline where this information can be found within the submission (e.g., sections or figure legends), or explain why this information doesn't apply to your submission:

No group allocation was used.

Additional data files ("source data")

- We encourage you to upload relevant additional data files, such as numerical data that are represented as a graph in a figure, or as a summary table
- Where provided, these should be in the most useful format, and they can be uploaded as "Source data" files linked to a main figure or table
- Include model definition files including the full list of parameters used
- Include code used for data analysis (e.g., R, MatLab)
- Avoid stating that data files are "available upon request"

Please indicate the figures or tables for which source data files have been provided:

Figure 2-Source Data 1 provides the full list of cross-linked peptides identified by mass-spectrometry in the 5-subunit full tRNA-LC sample on which Figure 2 and Figure 2-figure supplement 1A is based.

Figure 2-Source Data 2 provides the full list of cross-linked peptides identified by mass-spectrometry in the 4-subunit core tRNA-LC on which Figure 2-figure supplement 1B is based.
The MS raw data and the cross-linking results are available via ProteomeXchange with identifier PXD025662.

The structure factors and atomic model coordinates for the crystal structures are available via Protein Data Bank under accession codes 7P3A (N-terminal domain of CGI-99) and 7P3B (RTCB).
